# Effect of daratumumab on patients with high-risk cytogenetic multiple myeloma: a systematic review and meta-analysis of randomized controlled trials

**DOI:** 10.3389/fimmu.2026.1787690

**Published:** 2026-08-27

**Authors:** Liang Su, Minlan Ye, Bowen Peng, Lei Fan, Jie Wu

**Affiliations:** Guang’anmen Hospital, China Academy of Chinese Medical Sciences, Beijing, China

**Keywords:** daratumumab, meta-analysis, multiple myeloma, randomized controlled trials, systematic review

## Abstract

Although daratumumab has been reported to significantly improve the prognosis of multiple myeloma (MM), its efficacy in patients with cytogenetically high-risk MM (HRMM) remains unclear. This meta-analysis aims to evaluate the effect of daratumumab on progression-free survival (PFS) and overall survival (OS) in patients with cytogenetically HRMM. Compared to treatment without daratumumab, treatment with daratumumab showed significant PFS (HR = 0.58, *P* < 0.001) and OS (HR = 0.74, *P* = 0.017) benefit in patients with cytogenetically HRMM. Subgroup analyses revealed similar PFS benefits for newly diagnosed MM (HR = 0.63, *P* < 0.001), relapsed or refractory MM (HR = 0.53, *P* < 0.001), subcutaneous daratumumab (HR = 0.69, *P* = 0.038), and intravenous daratumumab (HR = 0.53, *P* < 0.001). Meanwhile, daratumumab significantly improved PFS in patients with revised high cytogenetic risk (HR = 0.51, *P* < 0.001). Another subgroup analysis revealed that daratumumab significantly improved PFS in both the one high-risk cytogenetic abnormality (HRCA) group (HR = 0.46, *P* < 0.001) and the isolated gain/amp(1q21) group (HR = 0.49, *P* < 0.001), while no significant improvement in PFS was observed in the group with more than or equal to two HRCAs (HR = 0.64, *P* = 0.212). However, sensitivity analysis showed that the beneficial OS is not robust. These results suggested that incorporating daratumumab into treatment regimens for patients with cytogenetic HRMM resulted in improved PFS. However, the effect of daratumumab on PFS may depend on HRCAs status. Additionally, the OS findings were suggestive of a potential benefit but require validation in future trials.

**Systematic review registration:**

https://www.crd.york.ac.uk/PROSPERO/, identifier CRD420251244272.

## Introduction

Multiple myeloma (MM) is a hematologic malignancy characterized by the clonal proliferation of malignant plasma cells, exhibiting highly heterogeneous clinical and biological features (1, 2). Cytogenetic abnormalities are key determinants of patient prognosis (3). According to the International Myeloma Working Group consensus, high-risk cytogenetic status is defined by the presence of at least one of the following abnormalities: t(4;14), t(14;16), or del(17p) (4, 5). Patients with MM who carry these high-risk cytogenetic abnormalities (HRCAs) typically respond poorly to conventional immunomodulatory therapies and generally have an unfavorable prognosis (6, 7). Daratumumab is the first-in-class IgG1κ monoclonal antibody targeting CD38, exerting antitumor effects through multiple mechanisms (8, 9). Daratumumab-based combination regimens have been proven to significantly improve progression-free survival (PFS) in both newly diagnosed MM (NDMM) and relapsed or refractory MM (RRMM) patients (10, 11). However, high-risk cytogenetic MM is a relatively rare cancer, and current evidence regarding the efficacy of daratumumab in such patients remains limited (12).

Previous meta-analyses have examined the effect of daratumumab on PFS in patients with high-risk cytogenetic MM (13, 14). However, results lack statistical consistency, potentially due to variations in the number of included studies. Additionally, results from different follow-up periods may exhibit statistical differences. Specifically, longer follow-up periods facilitate more accurate results. For instance, initial analysis from the CANDOR study showed that the PFS benefit in the high-risk cytogenetic MM group did not reach statistical significance (15). However, after an additional 11 months of follow-up, this benefit showed statistical significance (16). In recent years, several key Phase III trials have published long-term follow-up data (16–19), providing more mature and reliable evidence for the long-term efficacy of daratumumab, including its impact on high-risk populations. Additionally, as high-risk cytogenetic classification systems for MM have evolved, gain/amp(1q21) and t (14, 20) have increasingly been recognized as HRCAs (4, 20, 21). Among these, gain/amp(1q21) is one of the most common cytogenetic abnormalities in MM (22).

In summary, advances in high-risk cytogenetic MM research provide the foundation for updating meta-analysis. Therefore, this study aims to integrate existing randomized controlled trials (RCTs) for a systematic review and meta-analysis, thereby providing evidence for the clinical application of daratumumab in patients with high-risk cytogenetic MM. Beyond investigating its effect on PFS and overall survival (OS), this study also explores efficacy in specific subgroups, such as MM subtypes and revised cytogenetic risk stratification.

## Methods

This study adheres to the Preferred Reporting Items for Systematic Reviews and Meta-Analyses (PRISMA) guidelines (PROSPERO registration number: CRD420251244272) (23).

### Search strategy

To comprehensively identify relevant literature, we searched the following databases: PubMed, Embase, and the Cochrane Library. The search timeframe was set from database inception to 1 December 2025, and an updated search was conducted on 1 August 2026. The search strategy employed a combination of medical subject headings and free-text terms, and was restricted to English-language publications. Details of the literature search strategy are presented in **Supplementary Appendix 1**. All eligible studies were independently assessed by two reviewers (LS and MY). Disagreements were resolved by another reviewer (JW).

### Selection of studies

The inclusion criteria were as follows: (1) Study type: RCTs. (2) Population: patients diagnosed with MM and classified as having high-risk cytogenetic status. Based on relevant studies, high-risk cytogenetic status in this study was defined by the presence of at least one of the following HRCAs: t(4;14), t(14;16), or del(17p). In the revised definition, gain/amp(1q21) and t(14;20) were additionally considered HRCAs (4, 20). (3) Intervention: The control group received a standard therapy regimen without daratumumab; the intervention group received a combination regimen containing daratumumab. (4) Outcomes: PFS or OS. The exclusion criteria were as follows: (1) Conference abstracts with insufficient information, and duplicate publications. (2) Studies that did not report outcomes according to HRCAs status. (3) When multiple publications reported data from the same trial, only the data with the longest available follow-up were used for each endpoint. For trials involving a second randomization, only data from the first randomization were included.

### Data extraction

Literature management was conducted using EndNote software. The selection process will be performed independently by LS and MY, beginning with screening titles and abstracts, followed by full-text review. Any disagreements will be resolved through discussion or consultation with JW. Data extraction will utilize a pre-designed standardized form. Extracted information will include: basic study information (author, publication year, and trial name), patient characteristics (sample, MM subtypes, and HRCAs types), intervention and control regimens, follow-up duration, and data for outcome measures.

### Risk-of-bias assessment

The risk of bias for included RCTs was assessed independently by LS and MY using the Cochrane Collaboration’s recommended risk of bias assessment tool (24). This tool covers the following domains: random sequence generation, allocation concealment, blinding of participants and personnel, blinding of outcome assessment, incomplete outcome data, selective reporting, and other bias. For each study, the judgment for each domain was categorized as “low risk, “ “unclear risk, “ or “high risk.” Any disagreements arising during the assessment were resolved through consensus discussion or by consulting with JW.

### Data synthesis and analysis

The hazard ratio (HR) and its 95% confidence interval (CI) were used as the effect measure. Since results from random-effects models are more conservative, we employed a random-effects model for the meta-analysis to evaluate the effect of daratumumab on PFS and OS in patients with high-risk cytogenetic MM (25). Heterogeneity among the studies was assessed using Cochran’s Q test and the I² statistic. Sensitivity analysis was conducted using the leave-one-out method to evaluate the robustness of the pooled results. Subgroup analyses were performed based on MM subtypes, daratumumab administration methods, follow-up duration, and revised cytogenetic risk status. If ten or more studies were included, funnel plots along with Egger’s and Begg’s tests were used to quantitatively assess publication bias (26). All statistical analyses were performed using Stata software (version 16.0). A *P*-value of <0.05 was considered statistically significant.

## Results

### Search results and study characteristics

**Figure 1:**
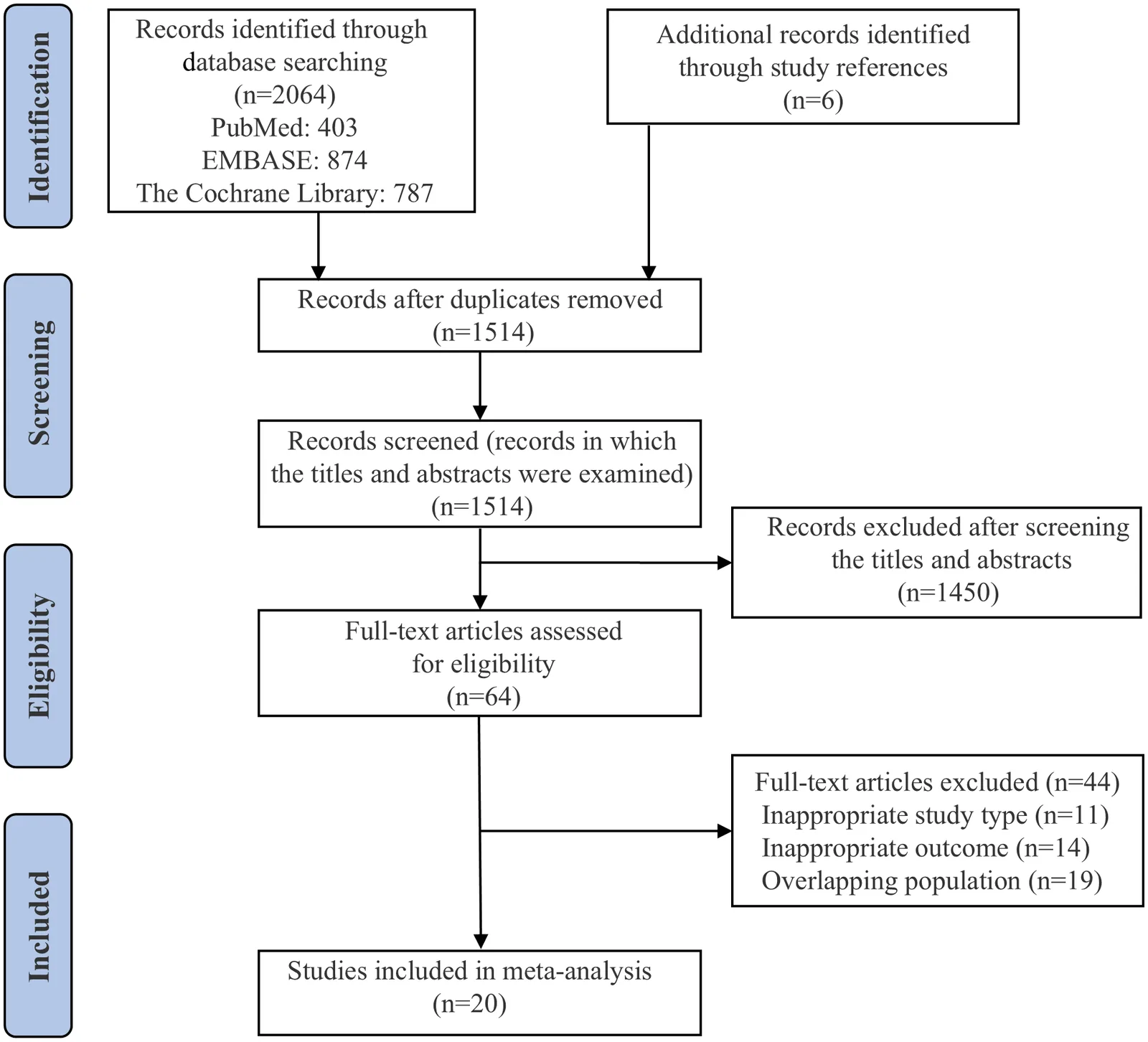
Flow diagram for the study selection process.

A total of 2, 064 publications were initially identified by the literature searches, of which 64 were reviewed in full. Ultimately, 20 publications involving 15 trials were included (11, 16–19, 27–41) (**Figure 1**; **Supplementary Appendix 2**). **Table 1** presents the characteristics of the included studies. These studies were conducted between 2018 and 2025, with follow-up periods ranging from 16.5 to 86.7 months. PFS was investigated in 15 RCTs (11, 16–19, 27, 28, 31, 34–39, 41), and OS was investigated in 5 RCTs (29, 30, 32, 33, 40). Regarding MM subtypes, eleven trials evaluated daratumumab in patients with NDMM (11, 17, 27, 29, 34–36, 38–41), and nine trials evaluated daratumumab in patients with RRMM (16, 18, 19, 28, 30–33, 37). Four trials reported outcomes based on revised definitions of cytogenetic risk (17, 34, 38, 39). The HRCAs reported in the included studies are summarized in **Supplementary Table 1**.

**Table 1:** Baseline characteristics of the included randomized controlled trials.

Study	Location	Trials	Number	MM type	Treatment	Method	Follow-up (months)	Outcome
Bahlis et al., 2025 (37)	Canada	LYNX	26	RRMM	D-Kd/Kd	SC	16.8	PFS
Chari et al., 2024 (34)	USA	GRIFFIN	79	NDMM	D-RVd/RVd	IV	49.6	PFS
Dimopoulos et al., 2021 (28)	Greece	APOLLO	74	RRMM	D-Pd/Pd	SC or IV	16.9	PFS
Dimopoulos et al., 2023 (30)	Greece	POLLUX	70	RRMM	D-Rd/Rd	IV	79.7	OS
Facon et al., 2021 (29)	France	MAIA	92	NDMM	D-Rd/Rd	IV	56.2	OS
Foster et al., 2025 (38)	USA	AURIGA	62	NDMM	D-R/R	SC	33.2	PFS
Fu et al., 2023 (31)	China	LEPUS	73	RRMM	D-Vd/Vd	SC or IV	25.1	PFS
Fu et al., 2025 (39)	China	OCTANS	122	NDMM	D-VMP/VMP	IV	41.2	PFS
Kaufman et al., 2020 (19)	USA	POLLUX	70	RRMM	D-Rd/Rd	IV	44.3	PFS
Mateos et al., 2018 (11)	Spain	ALCYONE	98	NDMM	D-VMP/VMP	IV	16.5	PFS
Mateos et al., 2025 (40)	Spain	ALCYONE	98	NDMM	D-VMP/VMP	IV	86.7	OS
Mollee et al., 2024 (35)	Australia	AMaRC 03-16	19	NDMM	D-VCd/VCd	IV	44.7	PFS
Moreau et al., 2019 (27)	France	CASSIOPEIA	168	NDMM	D-VTd/VTd	IV	18.8	PFS
Moreau et al., 2025 (17)	France	MAIA	308	NDMM	D-Rd/Rd	IV	64.5	PFS
Sonneveld et al., 2023 (32)	Netherlands	CASTOR	75	RRMM	D-Vd/Vd	IV	72.6	OS
Sonneveld et al., 2024 (36)	Netherlands	PERSEUS	154	NDMM	D-VRd/VRd	SC	47.5	PFS
Usmani et al., 2022 (16)	USA	CANDOR	74	RRMM	D-Kd/Kd	IV	27.8	PFS
Usmani et al., 2023 (33)	USA	CANDOR	74	RRMM	D-Kd/Kd	IV	50.6	OS
Usmani et al., 2025 (41)	USA	CEPHEUS	52	NDMM	D-VRd/VRd	SC	58.7	PFS
Weisel et al., 2020 (18)	Germany	CASTOR	75	RRMM	D-Vd/Vd	IV	40.0	PFS

HRCAs, high-risk cytogenetic abnormalities; IV, intravenous; MM, multiple myeloma; NDMM, newly diagnosed multiple myeloma; OS, overall survival; PFS, progression-free survival; RRMM, relapsed or refractory multiple myeloma; SC, subcutaneous; D, daratumumab; Kd, carfilzomib and dexamethasone; RVd, lenalidomide, bortezomib, and dexamethasone; Pd, pomalidomide and dexamethasone; Rd, lenalidomide and dexamethasone; R, lenalidomide; Vd, bortezomib and dexamethasone; VMP, bortezomib, melphalan, and prednisone; VCD, bortezomib, cyclophosphamide, and dexamethasone; VTd, bortezomib, thalidomide, and dexamethasone; VRd, bortezomib, lenalidomide, and dexamethasone.

### Risk-of-bias assessment

**Supplementary Figure 1** shows the results of the RoB assessment. Regarding the individual risks of bias, all included studies had a low risk of selection bias. However, they had a high risk of bias in terms of blinding of participants and personnel. Sixteen trials were considered to have a low risk of bias in the blinding of outcome assessment, while the remaining four trials were considered to have an unclear risk of bias. All included studies reported outcomes based on the intent-to-treat population and provided registration numbers, thus being assessed as having low risk of attrition bias and reporting bias. Regarding other bias, fourteen trials were considered to have a low risk of other bias, while the remaining six trials were considered to have an unclear risk.

### Effect of daratumumab on PFS

**Figure 2:**
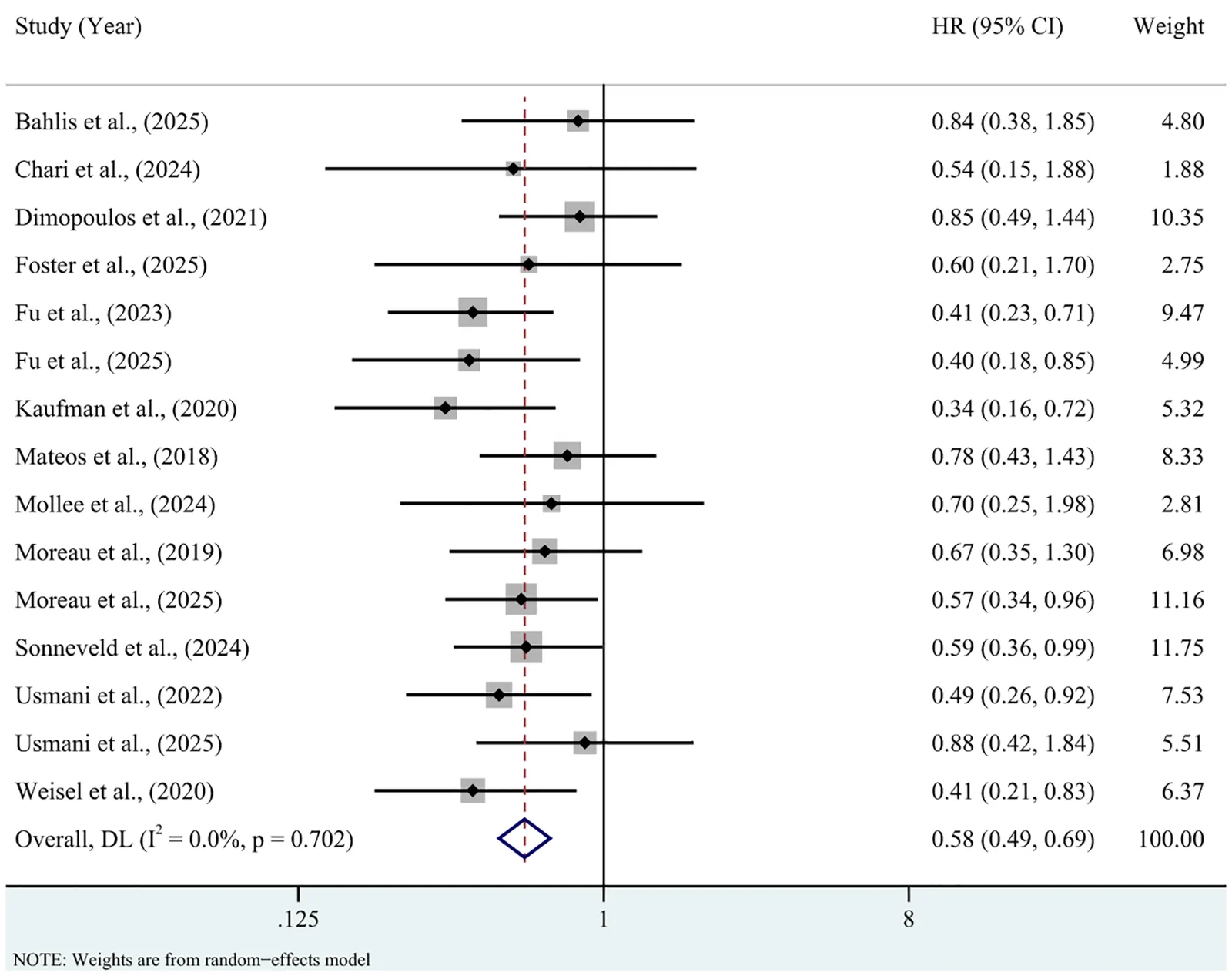
Forest plot for the effect of daratumumab on progression-free survival in patients with high-risk cytogenetic multiple myeloma.

In our meta-analysis incorporating 15 RCTs, daratumumab demonstrated a statistically significant PFS benefit, with an HR of 0.58 (95% CI = 0.49, 0.69, *P* < 0.001; *I*^2^ = 0%) (**Figure 2**). Furthermore, sensitivity analysis revealed that sequentially removing individual studies did not significantly alter the overall PFS results (**Supplementary Figure 2**).

**Figure 3:**
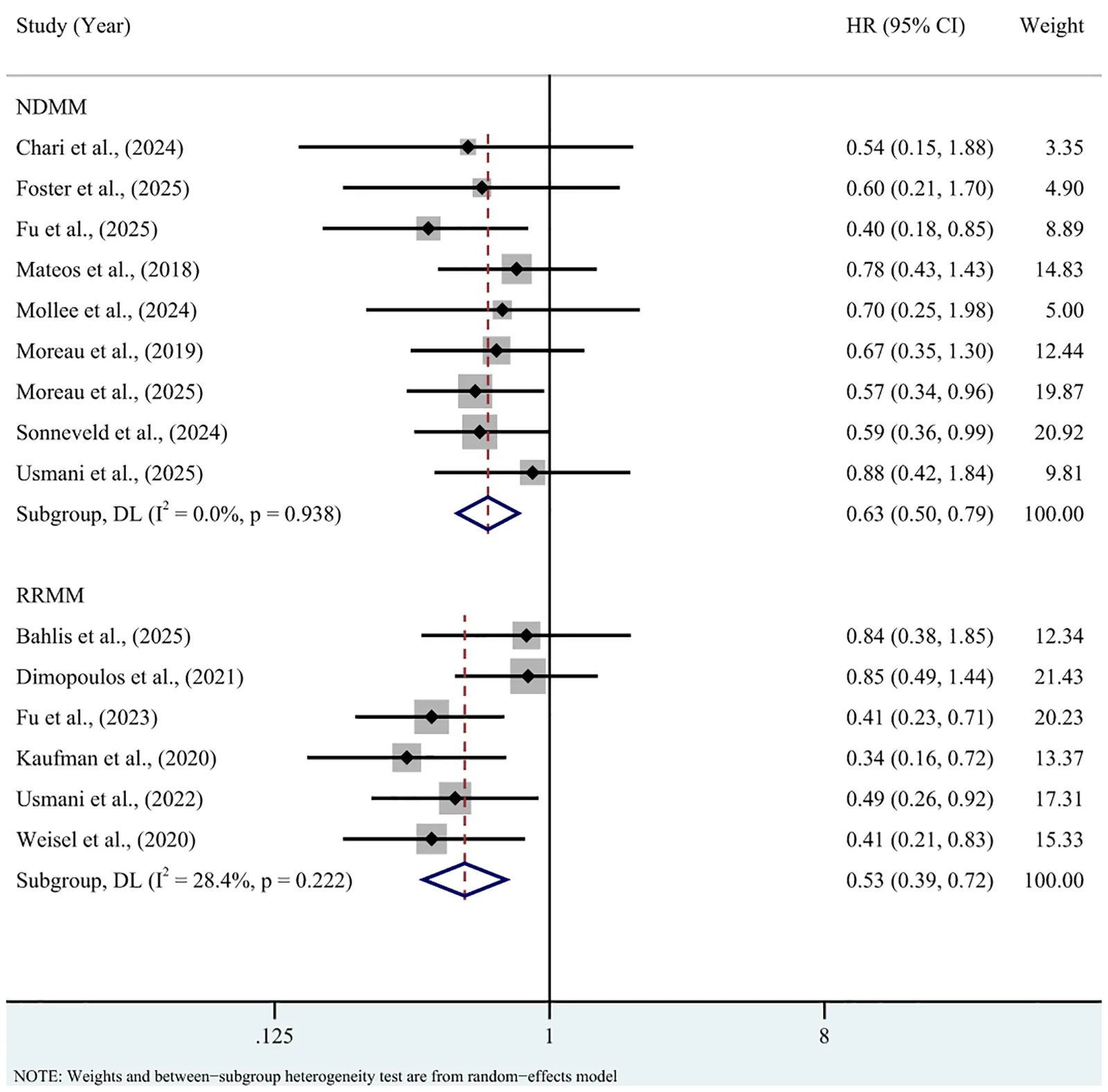
Forest plot for the subgroup analysis according to multiple myeloma subtypes (Progression-free survival). NDMM: newly diagnosed multiple myeloma, RRMM: relapsed or refractory multiple myeloma.

For subgroup analysis according to the MM subtypes, the finding was consistent with the overall results (**Figure 3**). Nine studies evaluated the effect of daratumumab in patients with NDMM, demonstrating a statistically significant PFS benefit (HR = 0.63; 95% CI = 0.50, 0.79, *P* < 0.001; *I*^2^ = 0%). Six studies evaluating the effects of daratumumab in patients with RRMM demonstrated a similar result (HR = 0.53; 95% CI = 0.39, 0.72, *P* < 0.001; *I*^2^ = 28.4%). According to the subgroup analysis based on daratumumab administration methods, the meta-analysis revealed that both subcutaneous (HR = 0.69; 95% CI = 0.49, 0.98, *P* = 0.038; *I*^2^ = 0%) and intravenous (HR = 0.53; 95% CI = 0.42, 0.68, *P* < 0.001; *I*^2^ = 0%) administration of daratumumab showed statistically significant PFS benefits (**Supplementary Figure 3**). According to the subgroup analysis stratified by follow-up duration, the meta-analysis revealed that daratumumab provided a statistically significant PFS benefit both in studies with follow-up shorter than the mean follow-up duration of the included studies (HR = 0.64; 95% CI = 0.50, 0.81, *P* < 0.001; *I*^2^ = 0%) and in those with follow-up longer than the mean follow-up duration (HR = 0.53; 95% CI = 0.42, 0.68, *P* < 0.001; *I*^2^ = 0%) (**Supplementary Figure 4**).

**Figure 4:**
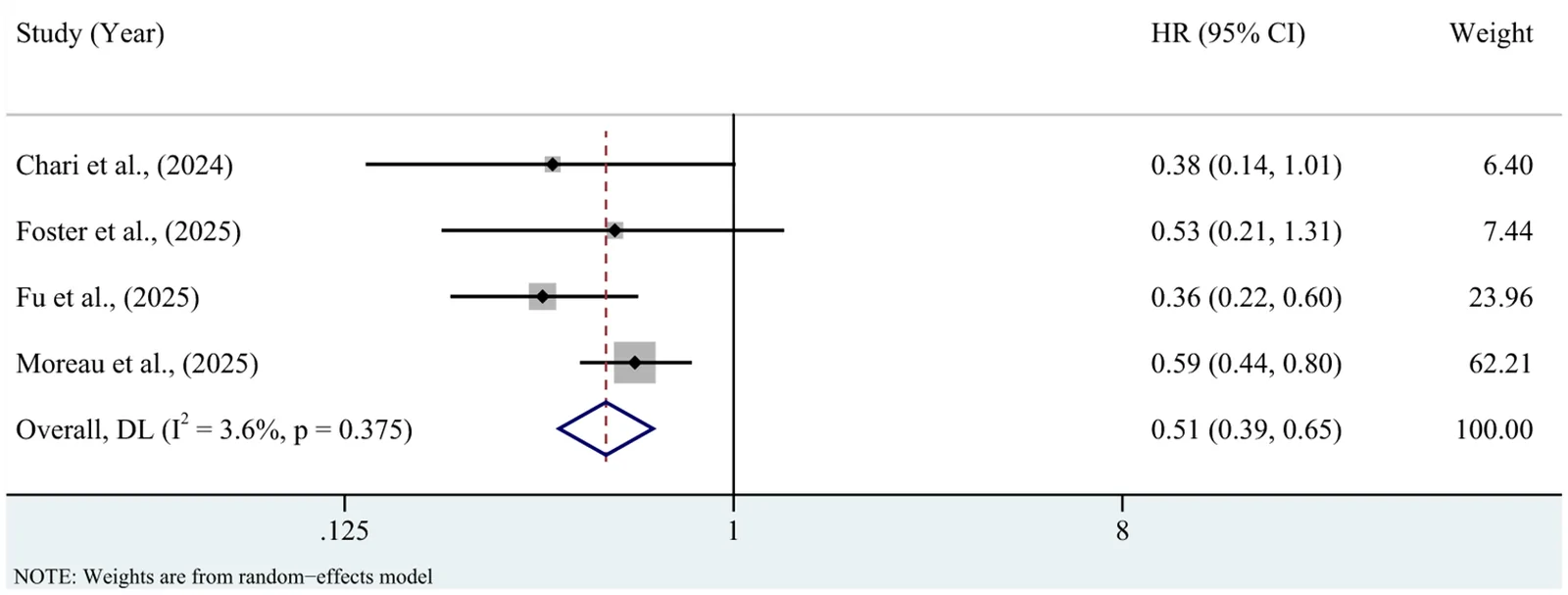
Forest plot for the effect of daratumumab on progression-free survival in multiple myeloma patients with revised high cytogenetic risk.

**Figure 5:**
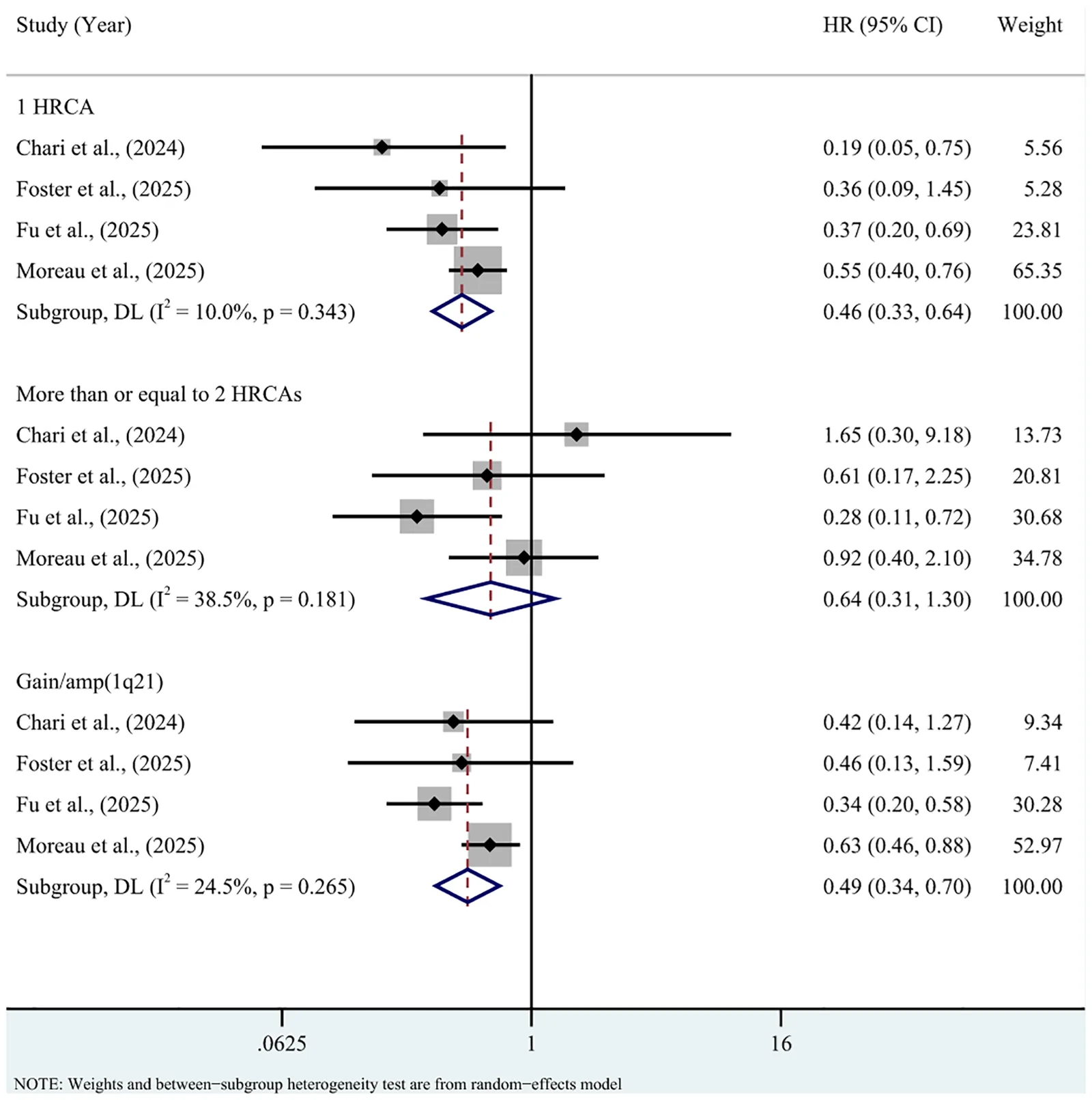
Forest plot for the subgroup analysis according to cytogenetic classification (Progression-free survival). HRCA: high-risk cytogenetic abnormality.

Four studies reported the effect of daratumumab on patients with revised cytogenetic risk. Pooled results demonstrated that daratumumab significantly improved PFS in patients with revised high cytogenetic risk (HR = 0.51; 95% CI = 0.39, 0.65, *P* < 0.001; *I*^2^ = 3.6%) (**Figure 4**). Sensitivity analysis revealed that sequentially removing individual studies did not significantly alter the overall results (**Supplementary Figure 5**). Subgroup analysis based on cytogenetic classification revealed that daratumumab significantly improved PFS in both the one HRCA group (HR = 0.46; 95% CI = 0.33, 0.64, *P* < 0.001; *I*^2^ = 10%) and the isolated gain/amp(1q21) group (HR = 0.49; 95% CI = 0.34, 0.7, *P* < 0.001; *I*^2^ = 24.5%), while no significant improvement in PFS was observed in the group with more than or equal to two HRCAs (HR = 0.64; 95% CI = 0.31, 1.3, *P* = 0.212; *I*^2^ = 38.5%) (**Figure 5**).

### Effect of daratumumab on OS

**Figure 6:**
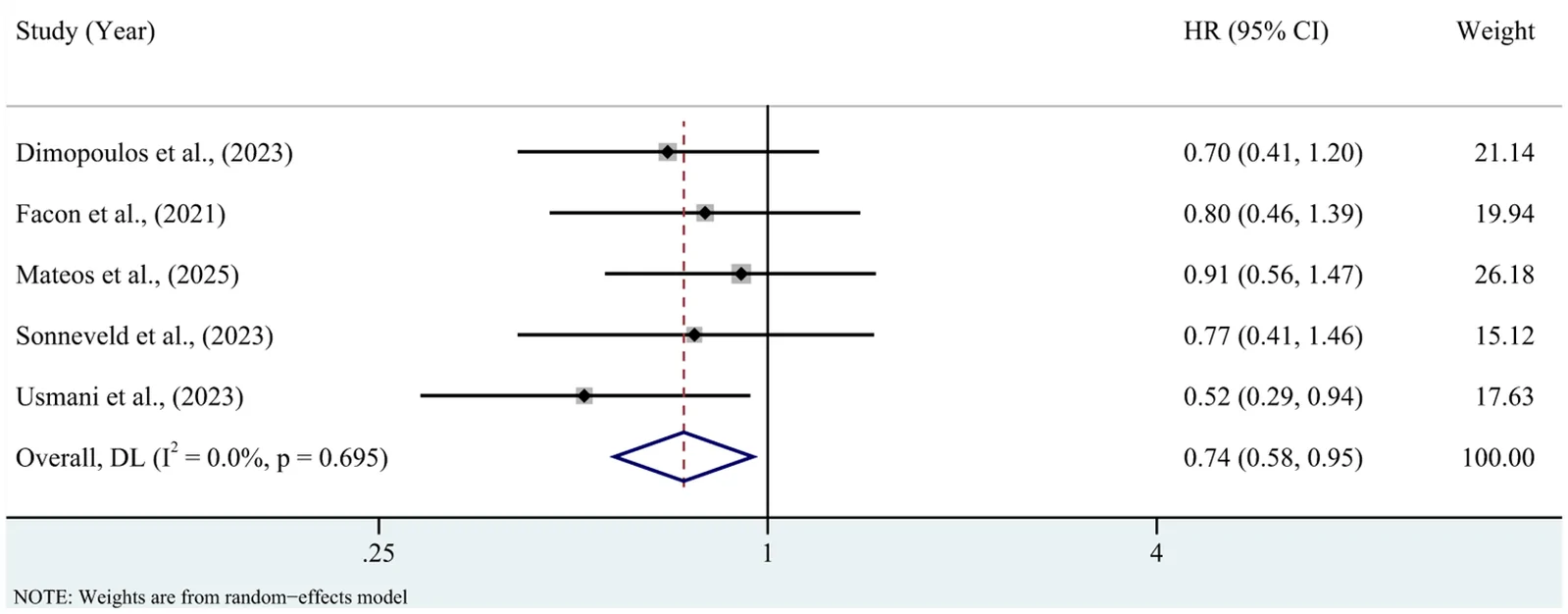
Forest plot for the effect of daratumumab on overall survival in patients with high-risk cytogenetic multiple myeloma.

Five studies reported the effect of daratumumab on OS. The pooled HR estimated by a random-effects model was 0.74 (95% CI = 0.58, 0.95, *P* = 0.017), with no significant evidence of heterogeneity between studies (*I*^2^ = 0%) (**Figure 6**). However, sensitivity analysis showed that this result lacked statistical benefit when Usmani SZ’s study was removed (**Supplementary Figure 6**).

Subgroup analysis based on MM subtypes is shown in **Supplementary Figure 7**. Three studies evaluated the effect of daratumumab in patients with RRMM, demonstrating a statistically significant OS benefit (HR = 0.65; 95% CI = 0.47, 0.91, *P* = 0.013; *I*^2^ = 0%). Only two studies evaluated the effect of daratumumab in patients with NDMM, and the pooled result showed no statistical significance (HR = 0.86; 95% CI = 0.60, 1.24, *P* = 0.419; *I*^2^ = 0%).

### Publication bias

Only trials evaluating the effect of daratumumab on PFS numbered more than 10; thus, a funnel plot was performed to assess publication bias. As shown in **Supplementary Figure 8**, there was no obvious asymmetry in the funnel plot. Furthermore, Egger’s test (*P* = 0.85; **Supplementary Figure 9**) and Begg’s test were not statistically significant (*P* = 0.692; **Supplementary Figure 10**).

## Discussion

This study evaluated the effect of daratumumab on PFS and OS in patients with high-risk cytogenetic MM. We found that incorporating daratumumab into MM treatment regimens improved PFS compared to regimens without daratumumab. Subgroup analysis showed that the PFS benefit was consistent with the overall finding for both NDMM and RRMM patients. A similar PFS benefit was also observed with both subcutaneous and intravenous administration of daratumumab. Furthermore, we evaluated the effect of daratumumab on PFS in patients with revised cytogenetic risk, with results potentially dependent on HRCAs status. Specifically, daratumumab significantly improved PFS in both the one HRCA group and the isolated gain/amp(1q21) group. However, no significant improvement in PFS was observed in the group with more than or equal to two HRCAs. This finding should be interpreted cautiously because the small number of studies may have limited the statistical power of this subgroup analysis. Additionally, the OS findings suggested a potential benefit; however, they were not statistically robust and require validation in additional trials with longer follow-up.

Over the past two decades, the emergence of novel therapies such as immunotherapy has revolutionized the treatment landscape for MM (42). Daratumumab, the first immunotherapeutic monoclonal antibody to target CD38, has been reported to significantly improve the prognosis of MM patients (43, 44). Notably, even with the introduction of daratumumab regimens, the prognosis for high-risk MM patients still lags behind that of standard-risk patients (43). Evidence has accumulated that identifying certain cytogenetic abnormalities is crucial for evaluating the prognosis of MM patients (45, 46). However, due to the small sample size of cytogenetic subgroups in previous studies, the association between patient prognosis and cytogenetic abnormalities has not been consistently investigated (43). In this context, meta-analysis can provide a more comprehensive investigation of survival outcomes by pooling data from various trials (47). A previously published meta-analysis has evaluated the efficacy of daratumumab on PFS in MM patients with high-risk cytogenetic factors (13). However, some studies included in this meta-analysis had not formally published their results at the time, limiting the ability to conduct stratified analyses. Furthermore, several studies now provide updated data from longer follow-up periods, which helped further elucidate the efficacy of daratumumab. Compared to the previous meta-analysis, this study included approximately three times as many trials. Our results revealed that incorporating daratumumab into MM regimens significantly improved PFS, with this benefit consistent across both NDMM and RRMM patients. On the other hand, daratumumab was initially approved for intravenous administration in the treatment of MM. Subsequently, the subcutaneous formulation gained attention for its superior convenience (48). Our findings showed that incorporating daratumumab into MM treatment regimens, either intravenously or subcutaneously, improved PFS compared to regimens without daratumumab. Additionally, statistically significant PFS benefits were observed in both subgroups stratified by follow-up duration. Nevertheless, given the observed variation in the effect sizes, studies with longer follow-up may yield more precise effect estimates. These findings have important clinical implications, supporting the disease control efficacy of the daratumumab treatment regimen.

MM is recognized as a complex disease, with most patients exhibiting recurrent primary chromosomal alterations at diagnosis. Concurrently, during disease progression, secondary genomic defects in sub-clones further drive tumor progression (49). The International Staging System (ISS) is the most widely used prognostic scoring system for risk stratification in MM patients (50). Specifically, the ISS incorporates del(17p), t (4;14), and t (14;16) as cytogenetic abnormalities, driving the shift towards cytogenetics-based risk stratification in MM (51). As understanding of the prognostic impact of genomic abnormalities in MM continues to evolve, subsequent studies have revealed that abnormalities in chromosome 1q21 represent one of the most common cytogenetic abnormalities, accounting for approximately 40% of newly diagnosed cases (51, 52). Importantly, chromosome 1q21 abnormalities have been reported to profoundly impact patient survival outcomes (53). In contrast, translocation t (14;16) is a rare primary genetic event, occurring in fewer than 5% of patients with NDMM (51). In this context, the revised ISS optimized the prognostic weighting of cytogenetic features by incorporating gain/amp(1q21) and excluding t(14;16) (51). Our findings showed that daratumumab significantly improved PFS in both the isolated gain/amp(1q21) group and the one HRCA group, potentially providing a reference for treatment options in patients with specific high-risk cytogenetics. However, high-risk cytogenetics are associated with high levels of clonal diversity and cellular proliferation, which may result in escape during daratumumab immunotherapy (43). Similarly, no statistically significant improvement in PFS was observed in the group with two or more HRCAs. However, this finding should be interpreted cautiously because the small number of studies may have limited the statistical power of this subgroup analysis. A recent study also reported that MM patients with more than two HRCAs exhibit the poorest prognosis; however, this finding requires confirmation in further trials (46). It is worth noting that the most effective drugs currently used to treat MM are not strictly targeted therapies, but rather act through pathways common to all plasma cells (54). Therefore, such patients may require specific targeted therapies.

Traditionally, PFS has been regarded as the primary endpoint for evaluating the efficacy of MM treatments in clinical trials (47). OS remains the ultimate endpoint in oncology clinical trials. We conducted a pooled analysis of OS in patients with high-risk cytogenetic MM, a critical endpoint that has not been addressed in earlier meta-analyses. Our results suggested a potential OS benefit associated with the incorporation of daratumumab into treatment regimens for patients with high-risk cytogenetic MM. This finding should be interpreted cautiously because only five studies were included in the pooled analysis, and the sensitivity analyses suggested that it was not statistically robust. However, as new therapies continue to improve patient outcomes, we cannot ignore the fact that assessing the OS superiority in MM clinical trials has become increasingly challenging (55). The significant improvement in PFS observed in our results supports further investigation into the potential for daratumumab regimens to improve OS.

Our findings provide cumulative evidence supporting the clinical application of daratumumab for high-risk cytogenetic MM. Meanwhile, the majority of results showed minimal heterogeneity, indicating a high level of consistency among the included studies and thereby enhancing the reliability of the pooled findings. Certainly, this study has limitations that cannot be denied. First, fluorescence *in situ* hybridization remains the gold standard for detecting genetic abnormalities in MM. However, specific practices may vary across different laboratories (56). Therefore, we cannot deny the confusion caused by the lack of standardization in testing methods. Second, there may be imbalances in patient characteristics between the groups included in the study. However, we cannot ignore the fact that high-risk cytogenetic MM is already a rare cancer, making targeted trials less realistic (12). Most research on this patient population primarily stems from subgroup analyses. This study exclusively included RCTs, all of which reported randomization methods and allocation concealment, thereby minimizing imbalances in patient characteristics. Third, we conducted subgroup analyses for MM subtypes, daratumumab administration methods, and revised cytogenetic risk stratification. However, other factors, such as extramedullary disease and lactate dehydrogenase levels, may also negatively impact prognosis. Due to limited data, we were unable to explore these issues. Future individual patient data meta-analysis may help elucidate such questions.

## Conclusions

In summary, incorporating daratumumab into treatment regimens for patients with high-risk cytogenetic MM resulted in improved PFS compared to regimens without daratumumab. However, the effect of daratumumab on PFS may depend on HRCAs status. The OS findings were suggestive of a potential benefit; however, they were not statistically robust and require validation in additional trials with longer follow-up.

## Data Availability

Publicly available datasets were analyzed in this study. This data can be found here: The data that support the findings of this study are available from the corresponding author upon reasonable request.

## References

[1] Kumar SK Rajkumar SV . The multiple myelomas - current concepts in cytogenetic classification and therapy. Nat Rev Clin Oncol. (2018) 15:409–21. doi: 10.1038/s41571-018-0018-y 29686421

[2] Luo M Qin L Li Y Mei Q Wu Q Feng X . Inflammatory markers from routine blood tests predict survival in multiple myeloma: a systematic review and meta-analysis. Front Immunol. (2025) 16:1669878. doi: 10.3389/fimmu.2025.1669878 41246322 PMC12611959

[3] Palumbo A Avet-Loiseau H Oliva S Lokhorst HM Goldschmidt H Rosinol L . Revised International Staging System for multiple myeloma: a report from International Myeloma Working Group. J Clin Oncol. (2015) 33:2863–9. doi: 10.1200/jco.2015.61.2267 26240224 PMC4846284

[4] Sonneveld P Avet-Loiseau H Lonial S Usmani S Siegel D Anderson KC . Treatment of multiple myeloma with high-risk cytogenetics: a consensus of the International Myeloma Working Group. Blood. (2016) 127:2955–62. doi: 10.1182/blood-2016-01-631200 27002115 PMC4920674

[5] Fonseca R Bergsagel PL Drach J Shaughnessy J Gutierrez N Stewart AK . International Myeloma Working Group molecular classification of multiple myeloma: spotlight review. Leukemia. (2009) 23:2210–21. doi: 10.1038/leu.2009.174 19798094 PMC2964268

[6] Avet-Loiseau H Attal M Campion L Caillot D Hulin C Marit G . Long-term analysis of the Ifm 99 trials for myeloma: cytogenetic abnormalities [T(4;14), del(17p), 1q gains] play a major role in defining long-term survival. J Clin Oncol. (2012) 30:1949–52. doi: 10.1200/jco.2011.36.5726 22547600

[7] Moreau P Cavo M Sonneveld P Rosinol L Attal M Pezzi A . Combination of International Scoring System 3, high lactate dehydrogenase, and T(4;14) and/or del(17p) identifies patients with multiple myeloma (Mm) treated with front-line autologous stem-cell transplantation at high risk of early Mm progression-related death. J Clin Oncol. (2014) 32:2173–80. doi: 10.1200/jco.2013.53.0329 24888806 PMC4879712

[8] Blair HA . Daratumumab: a review in relapsed and/or refractory multiple myeloma. Drugs. (2017) 77:2013–24. doi: 10.1007/s40265-017-0837-7 29127588

[9] van de Donk N Richardson PG Malavasi F . Cd38 antibodies in multiple myeloma: back to the future. Blood. (2018) 131:13–29. doi: 10.1182/blood-2017-06-740944 29118010

[10] Dimopoulos MA Oriol A Nahi H San-Miguel J Bahlis NJ Usmani SZ . Daratumumab, lenalidomide, and dexamethasone for multiple myeloma. N Engl J Med. (2016) 375:1319–31. doi: 10.1056/NEJMoa1607751 27705267

[11] Mateos MV Dimopoulos MA Cavo M Suzuki K Jakubowiak A Knop S . Daratumumab plus bortezomib, melphalan, and prednisone for untreated myeloma. N Engl J Med. (2018) 378:518–28. doi: 10.1056/NEJMoa1714678 29231133

[12] Lancman G Moshier E Cho HJ Parekh S Richard S Richter J . Trial designs and endpoints for immune therapies in multiple myeloma. Am J Hematol. (2023) 98:S35–45. doi: 10.1002/ajh.26753 36200130

[13] Giri S Grimshaw A Bal S Godby K Kharel P Djulbegovic B . Evaluation of daratumumab for the treatment of multiple myeloma in patients with high-risk cytogenetic factors: a systematic review and meta-analysis. JAMA Oncol. (2020) 6:1759–65. doi: 10.1001/jamaoncol.2020.4338 32970151 PMC7516804

[14] Chong LL Soon YY Soekojo CY Ooi M Chng WJ de Mel S . Daratumumab-based induction therapy for multiple myeloma: a systematic review and meta-analysis. Crit Rev Oncol Hematol. (2021) 159:103211. doi: 10.1016/j.critrevonc.2020.103211 33387628

[15] Dimopoulos M Quach H Mateos MV Landgren O Leleu X Siegel D . Carfilzomib, dexamethasone, and daratumumab versus carfilzomib and dexamethasone for patients with relapsed or refractory multiple myeloma (Candor): results from a randomised, multicentre, open-label, phase 3 study. Lancet. (2020) 396:186–97. doi: 10.1016/s0140-6736(20)30734-0 32682484

[16] Usmani SZ Quach H Mateos MV Landgren O Leleu X Siegel D . Carfilzomib, dexamethasone, and daratumumab versus carfilzomib and dexamethasone for patients with relapsed or refractory multiple myeloma (Candor): updated outcomes from a randomised, multicentre, open-label, phase 3 study. Lancet Oncol. (2022) 23:65–76. doi: 10.1016/s1470-2045(21)00579-9 34871550

[17] Moreau P Facon T Usmani SZ Bahlis N Raje N Plesner T . Daratumumab plus lenalidomide/dexamethasone in untreated multiple myeloma: analysis of key subgroups of the Maia study. Leukemia. (2025) 39:710–9. doi: 10.1038/s41375-024-02506-1 39815052

[18] Weisel K Spencer A Lentzsch S Avet-Loiseau H Mark TM Spicka I . Daratumumab, bortezomib, and dexamethasone in relapsed or refractory multiple myeloma: subgroup analysis of Castor based on cytogenetic risk. J Hematol Oncol. (2020) 13:115. doi: 10.1186/s13045-020-00948-5 32819447 PMC7439722

[19] Kaufman JL Dimopoulos MA White D Benboubker L Cook G Leiba M . Daratumumab, lenalidomide, and dexamethasone in relapsed/refractory myeloma: a cytogenetic subgroup analysis of Pollux. Blood Cancer J. (2020) 10:111. doi: 10.1038/s41408-020-00375-2 33149130 PMC7643179

[20] Voorhees PM Sborov DW Laubach J Kaufman JL Reeves B Rodriguez C . Addition of daratumumab to lenalidomide, bortezomib, and dexamethasone for transplantation-eligible patients with newly diagnosed multiple myeloma (Griffin): final analysis of an open-label, randomised, phase 2 trial. Lancet Haematol. (2023) 10:e825–37. doi: 10.1016/S2352-3026(23)00217-X 37708911

[21] Pang L Zhou M Chen C Qin P Yan S Dong X . Rapid complete remission after one cycle of isatuximab-based quadruplet regimen in 1q21-positive primary plasma cell leukemia: a case report. Front Immunol. (2026) 17:1815111. doi: 10.3389/fimmu.2026.1815111 42183230 PMC13194512

[22] Weinhold N Salwender HJ Cairns DA Raab MS Waldron G Blau IW . Chromosome 1q21 abnormalities refine outcome prediction in patients with multiple myeloma - a meta-analysis of 2,596 trial patients. Haematologica. (2021) 106:2754–8. doi: 10.3324/haematol.2021.278888 34092058 PMC8485656

[23] Page MJ McKenzie JE Bossuyt PM Boutron I Hoffmann TC Mulrow CD . The Prisma 2020 statement: an updated guideline for reporting systematic reviews. BMJ (Clin Res Ed). (2021) 372:n71. doi: 10.1136/bmj.n71 33782057 PMC8005924

[24] Higgins JP Altman DG Gøtzsche PC Jüni P Moher D Oxman AD . The Cochrane Collaboration's tool for assessing risk of bias in randomised trials. BMJ. (2011) 343:d5928. doi: 10.1136/bmj.d5928 22008217 PMC3196245

[25] Su L Xu C Huang H Zhang P Wang J Ouyang X . Effects of tumor necrosis factor-alpha inhibitors on lipid profiles in patients with psoriasis: a systematic review and meta-analysis. Front Immunol. (2024) 15:1354593. doi: 10.3389/fimmu.2024.1354593 38500874 PMC10944886

[26] Su L Wang F Wang Y Qin C Yang X Ye J . Circulating biomarkers of oxidative stress in people with acne vulgaris: a systematic review and meta-analysis. Arch Dermatol Res. (2024) 316:105. doi: 10.1007/s00403-024-02840-5 38489064

[27] Moreau P Attal M Hulin C Arnulf B Belhadj K Benboubker L . Bortezomib, thalidomide, and dexamethasone with or without daratumumab before and after autologous stem-cell transplantation for newly diagnosed multiple myeloma (Cassiopeia): a randomised, open-label, phase 3 study. Lancet (London England). (2019) 394:29–38. doi: 10.1016/S0140-6736(19)31240-1 31171419

[28] Dimopoulos MA Terpos E Boccadoro M Delimpasi S Beksac M Katodritou E . Daratumumab plus pomalidomide and dexamethasone versus pomalidomide and dexamethasone alone in previously treated multiple myeloma (Apollo): an open-label, randomised, phase 3 trial. Lancet Oncol. (2021) 22:801–12. doi: 10.1016/S1470-2045(21)00128-5 34087126

[29] Facon T Kumar SK Plesner T Orlowski RZ Moreau P Bahlis N . Daratumumab, lenalidomide, and dexamethasone versus lenalidomide and dexamethasone alone in newly diagnosed multiple myeloma (Maia): overall survival results from a randomised, open-label, phase 3 trial. Lancet Oncol. (2021) 22:1582–96. doi: 10.1016/S1470-2045(21)00466-6 34655533

[30] Dimopoulos MA Oriol A Nahi H San-Miguel J Bahlis NJ Usmani SZ . Overall survival with daratumumab, lenalidomide, and dexamethasone in previously treated multiple myeloma (Pollux): a randomized, open-label, phase iii trial. J Clin Oncol. (2023) 41:1590–9. doi: 10.1200/JCO.22.00940 36599114 PMC10022849

[31] Fu W Li W Hu J An G Wang Y Fu C . Daratumumab, bortezomib, and dexamethasone versus bortezomib and dexamethasone in Chinese patients with relapsed or refractory multiple myeloma: updated analysis of Lepus. Clin Lymphoma Myeloma Leukemia. (2023) 23:e51–8. doi: 10.1016/j.clml.2022.10.007 36402700

[32] Sonneveld P Chanan-Khan A Weisel K Nooka AK Masszi T Beksac M . Overall survival with daratumumab, bortezomib, and dexamethasone in previously treated multiple myeloma (Castor): a randomized, open-label, phase iii trial. J Clin Oncol. (2023) 41:1600–9. doi: 10.1200/JCO.21.02734 36413710 PMC10022857

[33] Usmani SZ Quach H Mateos MV Landgren O Leleu X Siegel D . Final analysis of carfilzomib, dexamethasone, and daratumumab vs carfilzomib and dexamethasone in the Candor study. Blood Adv. (2023) 7:3739–48. doi: 10.1182/bloodadvances.2023010026 37163358 PMC10368773

[34] Chari A Kaufman JL Laubach J Sborov DW Reeves B Rodriguez C . Daratumumab in transplant-eligible patients with newly diagnosed multiple myeloma: final analysis of clinically relevant subgroups in Griffin. Blood Cancer J. (2024) 14:107. doi: 10.1038/s41408-024-01088-6 38977707 PMC11231363

[35] Mollee P Reynolds J Janowski W Quach H Campbell P Gibbs S . Daratumumab, cyclophosphamide, bortezomib, and dexamethasone for transplant-ineligible myeloma: Amarc 03-16. Blood Adv. (2024) 8:3721–30. doi: 10.1182/bloodadvances.2023012539 38739707 PMC11296246

[36] Sonneveld P Dimopoulos MA Boccadoro M Quach H Ho PJ Beksac M . Daratumumab, bortezomib, lenalidomide, and dexamethasone for multiple myeloma. N Engl J Med. (2024) 390:301–13. doi: 10.1056/NEJMoa2312054 38084760

[37] Bahlis NJ Zonder J Karlin L Plesner T Paris L Wrobel T . Subcutaneous daratumumab plus carfilzomib and dexamethasone (D-Kd) versus carfilzomib and dexamethasone (Kd) in patients with relapsed/refractory multiple myeloma who received previous daratumumab treatment: Lynx study. Leukemia Lymphoma. (2025) 66:2685–96. doi: 10.1080/10428194.2025.2561117 41041907

[38] Foster L Anderson LD Chung A Chaulagain CP Pettijohn E Cowan AJ . Daratumumab plus lenalidomide maintenance in newly diagnosed multiple myeloma after transplant: Auriga subgroup analyses. Blood Cancer J. (2025) 15:154. doi: 10.1038/s41408-025-01355-0 41053004 PMC12500855

[39] Fu W Bang SM Huang H Kim K Li W An G . Daratumumab, bortezomib, melphalan, and prednisone versus bortezomib, melphalan, and prednisone alone in transplant-ineligible Asian patients with newly diagnosed multiple myeloma: final analysis of the phase 3 Octans study. Ann Hematol. (2025) 104:515–25. doi: 10.1007/s00277-024-05958-8 39227450 PMC11868237

[40] Mateos MV San-Miguel J Cavo M Suzuki K Jakubowiak A Knop S . Bortezomib, melphalan, and prednisone with or without daratumumab in transplant-ineligible patients with newly diagnosed multiple myeloma (Alcyone): final analysis of an open-label, randomised, multicentre, phase 3 trial. Lancet Oncol. (2025) 26:596–608. doi: 10.1016/S1470-2045(25)00018-X 40220771

[41] Usmani SZ Facon T Hungria V Bahlis NJ Venner CP Braunstein M . Daratumumab plus bortezomib, lenalidomide and dexamethasone for transplant-ineligible or transplant-deferred newly diagnosed multiple myeloma: the randomized phase 3 Cepheus trial. Nat Med. (2025) 31:1195–202. doi: 10.1038/s41591-024-03485-7 39910273 PMC12003169

[42] Chakraborty R Siddiqi R Willson G Gupta S Asghar N Husnain M . Impact of autologous transplantation on survival in patients with newly diagnosed multiple myeloma who have high-risk cytogenetics: a meta-analysis of randomized controlled trials. Cancer. (2022) 128:2288–97. doi: 10.1002/cncr.34211 35377484

[43] Yang Y Li Y Gu H Dong M Cai Z . Emerging agents and regimens for multiple myeloma. J Hematol Oncol. (2020) 13:150. doi: 10.1186/s13045-020-00980-5 33168044 PMC7654052

[44] Horenstein AL Frerichs KA Faini AC van de Donk N Malavasi F . Modulating Cd38 enzymatic activity during antibody-based immunotherapy in multiple myeloma: a basic science perspective. Front Immunol. (2026) 17:1769281. doi: 10.3389/fimmu.2026.1769281 42136641 PMC13168026

[45] Kasomva K Yadav K Janz S Dhakal B Rao S . Molecular and immunological determinants of long-term survival in multiple myeloma. Blood Adv. (2025) 9:5134–47. doi: 10.1182/bloodadvances.2025016829 40674709 PMC12550235

[46] Kaiser MF Sonneveld P Cairns DA Raab MS San-Miguel Izquierdo J Zhang R . Co-occurrence of cytogenetic abnormalities and high-risk disease in newly diagnosed and relapsed/refractory multiple myeloma. J Clin Oncol Off J Am Soc Clin Oncol. (2025) 43:2679–91. doi: 10.1200/JCO-24-01253 39965171 PMC12352566

[47] Souto Filho JTD Cantadori LO Crusoe EDQ Hungria V Maiolino A . Daratumumab-based quadruplet versus triplet induction regimens in transplant-eligible newly diagnosed multiple myeloma: a systematic review and meta-analysis. Blood Cancer J. (2025) 15:37. doi: 10.1038/s41408-025-01253-5 40082415 PMC11906644

[48] Usmani SZ Nahi H Legiec W Grosicki S Vorobyev V Spicka I . Final analysis of the phase iii non-inferiority Columba study of subcutaneous versus intravenous daratumumab in patients with relapsed or refractory multiple myeloma. Haematologica. (2022) 107:2408–17. doi: 10.3324/haematol.2021.279459 35354247 PMC9521240

[49] Clarke SE Fuller KA Erber WN . Chromosomal defects in multiple myeloma. Blood Rev. (2024) 64:101168. doi: 10.1016/j.blre.2024.101168 38212176

[50] Mosquera Orgueira A Gonzalez Perez MS Diaz Arias J Rosinol L Oriol A Teruel AI . Unsupervised machine learning improves risk stratification in newly diagnosed multiple myeloma: an analysis of the Spanish Myeloma Group. Blood Cancer J. (2022) 12:76. doi: 10.1038/s41408-022-00647-z 35468898 PMC9038663

[51] Zanwar S Rajkumar SV . Current risk stratification and staging of multiple myeloma and related clonal plasma cell disorders. Leukemia. (2025) 39:2610–7. doi: 10.1038/s41375-025-02654-y 40702148 PMC12589131

[52] Schmidt TM Fonseca R Usmani SZ . Chromosome 1q21 abnormalities in multiple myeloma. Blood Cancer J. (2021) 11:83. doi: 10.1038/s41408-021-00474-8 33927196 PMC8085148

[53] Ntanasis-Stathopoulos I Filippatos C Malandrakis P Koutoulidis V Trapali M Kastritis E . The impact of high-risk cytogenetics on treatment efficacy and outcomes of patients with relapsed/refractory multiple myeloma: A systematic review and meta-analysis of randomized controlled trials. Leukemia. (2025) 39:2226–36. doi: 10.1038/s41375-025-02677-5 40603652

[54] Kumar S Baizer L Callander NS Giralt SA Hillengass J Freidlin B . Gaps and opportunities in the treatment of relapsed-refractory multiple myeloma: Consensus recommendations of the Nci multiple myeloma steering committee. Blood Cancer J. (2022) 12:98. doi: 10.1038/s41408-022-00695-5 35768410 PMC9243011

[55] Landgren O Prior TJ Masterson T Heuck C Bueno OF Dash AB . Evidence meta-analysis: Evaluating minimal residual disease as an intermediate clinical end point for multiple myeloma. Blood. (2024) 144:359–67. doi: 10.1182/blood.2024024371 38768337 PMC11418064

[56] Lu X Andersen EF Banerjee R Eno CC Gonzales PR Kumar S . Guidelines for the testing and reporting of cytogenetic results for risk stratification of multiple myeloma: A report of the Cancer Genomics Consortium Plasma Cell Neoplasm Working Group. Blood Cancer J. (2025) 15:86. doi: 10.1038/s41408-025-01286-w 40533444 PMC12177088

