# Supplementary material for: Effect of daratumumab on patients with high-risk cytogenetic multiple myeloma: a systematic review and meta-analysis of randomized controlled trials
Source: Front Immunol. 2026 Aug 27;17:1787690. doi: 10.3389/fimmu.2026.1787690 (PMC13558208; doi:10.3389/fimmu.2026.1787690)
Supplement: Supplementary file 1 [file Table1.docx]

**Effect of Daratumumab on Patients with High-risk Cytogenetic Multiple Myeloma: A Systematic Review and Meta-Analysis of Randomized Controlled Trials**

Liang Su et al.

**“Online Supplementary Material”**

Supplemental Appendix 1:

Full search strategy: PubMed

**Searches**

#1 (("Multiple Myeloma"[Mesh] OR "Smoldering Multiple Myeloma"[Mesh]) OR "Monoclonal Gammopathy of Undetermined Significance"[Mesh]) OR (((((((((Multiple Myeloma[Title/Abstract]) OR (Smoldering Multiple Myeloma[Title/Abstract])) OR (Monoclonal Gammopathy of Undetermined Significance[Title/Abstract])) OR (Plasma Cell Myeloma[Title/Abstract])) OR (Myelomas, Multiple[Title/Abstract])) OR (Myeloma, Plasma-Cell[Title/Abstract])) OR (Cell Myeloma, Plasma[Title/Abstract])) OR (Kahler Disease[Title/Abstract])) OR (Myelomatosis[Title/Abstract]))

#2 "Daratumumab"[Title/Abstract] OR " Darzalex "[Title/Abstract] OR " humax-CD38 "[Title/Abstract]

#3 "randomized controlled trial"[Publication Type] OR "controlled clinical trial"[Publication Type] OR "randomized"[Title/Abstract] OR " randomised "[Title/Abstract] OR "randomly"[Title/Abstract] OR "random"[Title/Abstract] OR "comparative study"[Title/Abstract] OR "trial"[Title/Abstract] OR "single blind"[Title/Abstract] OR "double blind"[Title/Abstract] OR "triple blind"[Title/Abstract] OR "placebo"[Title/Abstract]

#4 #1 and #2 and #3

#5 limit #4 to English language

Supplemental Appendix 2: Full-text publications excluded with reason

| **Online Supplementary Reference** | **Reason for exclusion** |
| --- | --- |
| (1-11) | Inappropriate study type |
| (12-25) | Inappropriate outcome |
| (26-44) | Overlapping population |

Online Supplementary References

1.	Callander NS, Silbermann R, Kaufman JL, Godby KN, Laubach J, Schmidt TM, et al. Daratumumab-Based Quadruplet Therapy for Transplant-Eligible Newly Diagnosed Multiple Myeloma with High Cytogenetic Risk. *Blood cancer journal* (2024) 14(1):69. doi: 10.1038/s41408-024-01030-w.

2.	Costa LJ, Chhabra S, Medvedova E, Dholaria BR, Schmidt TM, Godby KN, et al. Daratumumab, Carfilzomib, Lenalidomide, and Dexamethasone with Minimal Residual Disease Response-Adapted Therapy in Newly Diagnosed Multiple Myeloma. *Journal of Clinical Oncology* (2022) 40(25):2901-12. doi: 10.1200/JCO.21.01935.

3.	Derman BA, Zonder J, Reece D, Cole C, Berdeja J, Stefka AT, et al. Phase 1/2 Study of Carfilzomib, Pomalidomide, and Dexamethasone with and without Daratumumab in Relapsed Multiple Myeloma. *Blood Advances* (2023) 7(19):5703-12. doi: 10.1182/bloodadvances.2022008866.

4.	Kaiser MF, Hall A, Walker K, Sherborne A, De Tute RM, Newnham N, et al. Daratumumab, Cyclophosphamide, Bortezomib, Lenalidomide, and Dexamethasone as Induction and Extended Consolidation Improves Outcome in Ultra-High-Risk Multiple Myeloma. *Journal of clinical oncology* (2023) 41(23):3945‐55. doi: 10.1200/JCO.22.02567.

5.	Kumar L, Melinkeri S, Ganesan P, Kumar J, Biswas G, Kilara N, et al. Daratumumab in Indian Patients with Relapsed and Refractory Multiple Myeloma: A Prospective, Multicenter, Phase Iv Study. *Future oncology (London, England)* (2024) 20(4):191-205. Epub 2023/12/20. doi: 10.2217/fon-2023-0842.

6.	Manier S, Lambert J, Hulin C, Macro M, Laribi K, Araujo C, et al. Safety and Efficacy of a Dexamethasone-Sparing Regimen with Daratumumab and Lenalidomide in Patients with Frailty and Newly Diagnosed Multiple Myeloma (Ifm2017-03): A Phase 3, Open-Label, Multicentre, Randomised, Controlled Trial. *The lancet Oncology* (2025) 26(10):1323‐33. doi: 10.1016/S1470-2045(25)00280-3.

7.	Mateos MV, Dimopoulos MA, Cavo M, Suzuki K, Knop S, Doyen C, et al. Daratumumab Plus Bortezomib, Melphalan, and Prednisone Versus Bortezomib, Melphalan, and Prednisone in Transplant-Ineligible Newly Diagnosed Multiple Myeloma: Frailty Subgroup Analysis of Alcyone. *Clinical lymphoma, myeloma & leukemia* (2021) 21(11):785‐98. doi: 10.1016/j.clml.2021.06.005.

8.	Suzuki T, Terada M, Machida R, Kataoka T, Ito Y, Kataoka K, et al. Randomized Phase Iii Study of Daratumumab Versus Bortezomib Plus Daratumumab as Maintenance Therapy after D-Mpb for Transplant-Ineligible Patients with Untreated Multiple Myeloma (Jcog1911, B-Dash Study). *Japanese journal of clinical oncology* (2023) 53(4):349‐54. doi: 10.1093/jjco/hyac198.

9.	Costa LJ, Bahlis NJ, Perrot A, Nooka AK, Lu J, Pawlyn C, et al. Teclistamab Plus Daratumumab in Relapsed or Refractory Multiple Myeloma. *New England journal of medicine* (2026) 394(8):739‐52. doi: 10.1056/NEJMoa2514663.

10.	Maclachlan KH, Tan CR, Shekarkhand T, Rueda C, Derkach A, Hashmi H, et al. Ims-Imwg 2025 Consensus Genomic Staging Predicts Outcomes with Daratumumab-Based Quadruplet Regimens for Ndmm. *Blood Advances* (2026) 10(8):2616-25. doi: 10.1182/bloodadvances.2025018537.

11.	Dimopoulos MA, Voorhees PM, Schjesvold F, Cohen YC, Hungria V, Sandhu I, et al. Daratumumab or Active Monitoring for High-Risk Smoldering Multiple Myeloma. *New England journal of medicine* (2025) 392(18):1777‐88. doi: 10.1056/NEJMoa2409029.

12.	Bahlis NJ, Siegel DS, Schiller GJ, Samaras C, Sebag M, Berdeja J, et al. Pomalidomide, Dexamethasone, and Daratumumab Immediately after Lenalidomide-Based Treatment in Patients with Multiple Myeloma: Updated Efficacy, Safety, and Health-Related Quality of Life Results from the Phase 2 Mm-014 Trial. *Leukemia & lymphoma* (2022) 63(6):1407-17. Epub 2022/02/09. doi: 10.1080/10428194.2022.2030477.

13.	Dimopoulos MA, Oriol A, Nahi H, San-Miguel J, Bahlis NJ, Usmani SZ, et al. Daratumumab, Lenalidomide, and Dexamethasone for Multiple Myeloma. *New England journal of medicine* (2016) 375(14):1319‐31. doi: 10.1056/NEJMoa1607751.

14.	Dimopoulos MA, Terpos E, Boccadoro M, Delimpasi S, Beksac M, Katodritou E, et al. Subcutaneous Daratumumab Plus Pomalidomide and Dexamethasone Versus Pomalidomide and Dexamethasone in Patients with Relapsed or Refractory Multiple Myeloma (Apollo): Extended Follow up of an Open-Label, Randomised, Multicentre, Phase 3 Trial. *The Lancet Haematology* (2023) 10(10):e813‐e24. doi: 10.1016/S2352-3026(23)00218-1.

15.	Facon T, Moreau P, Weisel K, Goldschmidt H, Usmani SZ, Chari A, et al. Daratumumab/Lenalidomide/Dexamethasone in Transplant-Ineligible Newly Diagnosed Myeloma: Maia Long-Term Outcomes: Multiple Myeloma, Gammopathies. *Leukemia* (2025). doi: 10.1038/s41375-024-02505-2.

16.	Fujisaki T, Ishikawa T, Takamatsu H, Suzuki K, Min CK, Lee JH, et al. Daratumumab Plus Bortezomib, Melphalan, and Prednisone in East Asian Patients with Non-Transplant Multiple Myeloma: Subanalysis of the Randomized Phase 3 Alcyone Trial. *Annals of hematology* (2019) 98(12):2805‐14. doi: 10.1007/s00277-019-03794-9.

17.	Mateos MV, Cavo M, Blade J, Dimopoulos MA, Suzuki K, Jakubowiak A, et al. Overall Survival with Daratumumab, Bortezomib, Melphalan, and Prednisone in Newly Diagnosed Multiple Myeloma (Alcyone): A Randomised, Open-Label, Phase 3 Trial. *Lancet (london, england)* (2020) 395(10218):132‐41. doi: 10.1016/S0140-6736(19)32956-3.

18.	Mateos MV, Paiva B, Cedena MT, Puig N, Sureda-Balari AM, de la Calle VG, et al. Induction Therapy with Bortezomib, Melphalan, and Prednisone Followed by Lenalidomide and Dexamethasone Versus Carfilzomib, Lenalidomide, and Dexamethasone with or without Daratumumab in Older, Fit Patients with Newly Diagnosed Multiple Myeloma (Gem-2017fit): A Phase 3, Open-Label, Multicentre, Randomised Clinical Trial. *The Lancet Haematology* (2025) 12(8):e588‐e98. doi: 10.1016/S2352-3026(25)00143-7.

19.	Palumbo A, Chanan-Khan A, Weisel K, Nooka AK, Masszi T, Beksac M, et al. Daratumumab, Bortezomib, and Dexamethasone for Multiple Myeloma. *New England journal of medicine* (2016) 375(8):754‐66. doi: 10.1056/NEJMoa1606038.

20.	Roussel M, Moreau P, Hebraud B, Laribi K, Jaccard A, Dib M, et al. Bortezomib, Thalidomide, and Dexamethasone with or without Daratumumab for Transplantation-Eligible Patients with Newly Diagnosed Multiple Myeloma (Cassiopeia): Health-Related Quality of Life Outcomes of a Randomised, Open-Label, Phase 3 Trial. *The Lancet Haematology* (2020) 7(12):e874‐e83. doi: 10.1016/S2352-3026(20)30356-2.

21.	Suzuki K, Dimopoulos MA, Takezako N, Okamoto S, Shinagawa A, Matsumoto M, et al. Daratumumab, Lenalidomide, and Dexamethasone in East Asian Patients with Relapsed or Refractory Multiple Myeloma: Subgroup Analyses of the Phase 3 Pollux Study. *Blood cancer journal* (2018) 8(4). doi: 10.1038/s41408-018-0071-x.

22.	Voorhees PM, Kaufman JL, Laubach J, Sborov DW, Reeves B, Rodriguez C, et al. Daratumumab, Lenalidomide, Bortezomib, and Dexamethasone for Transplant-Eligible Newly Diagnosed Multiple Myeloma: The Griffin Trial. *Blood* (2020) 136(8):936‐45. doi: 10.1182/blood.2020005288.

23.	Zhang YH, Xu F, Xu CQ, Zhang ZT, Jiao ZJ. Effect of Daratumumab Combined with Chemotherapy on Immune Function in Patients with Relapsed/Refractory Multiple Myeloma and Observation of Its Clinical Efficacy. *Pakistan journal of medical sciences* (2023) 39(1). doi: 10.12669/pjms.39.1.6667.

24.	Jamroziak K, Dytfeld D, Kubicki T, Dutka M, Wróbel T, Giannopoulos K, et al. Daratumumab or Active Observation for Asymptomatic Biochemical Relapse in Patients with Multiple Myeloma. *British journal of haematology* (2026) 208(4):1314‐23. doi: 10.1111/bjh.70373.

25.	Kumar S, Knopf B, Asmus E, Gertz M, Kapoor P, Hayman S, et al. Ixazomib, Cyclophosphamide, and Dexamethasone (Icd), with or without Daratumumab in Relapsed Multiple Myeloma. *Clinical Lymphoma, Myeloma and Leukemia* (2026). doi: 10.1016/j.clml.2026.05.004.

26.	Badros A, Foster L, Anderson LD, Chaulagain CP, Pettijohn E, Cowan AJ, et al. Daratumumab with Lenalidomide as Maintenance after Transplant in Newly Diagnosed Multiple Myeloma: The Auriga Study. *Blood* (2025) 145(3):300‐10. doi: 10.1182/blood.2024025746.

27.	Bahlis NJ, Dimopoulos MA, White DJ, Benboubker L, Cook G, Leiba M, et al. Daratumumab Plus Lenalidomide and Dexamethasone in Relapsed/Refractory Multiple Myeloma: Extended Follow-up of Pollux, a Randomized, Open-Label, Phase 3 Study. *Leukemia* (2020) 34(7):1875‐84. doi: 10.1038/s41375-020-0711-6.

28.	Bertamini L, Fokkema C, Rodriguez-Otero P, van Duin M, Terpos E, D'Agostino M, et al. Circulating Tumor Cells Predict Myeloma Outcomes in Patients Treated with Daratumumab, Bortezomib, Lenalidomide, and Dexamethasone. *Blood* (2025). doi: 10.1182/blood.2025030113.

29.	Corre J, Vincent L, Moreau P, Hebraud B, Hulin C, Béné MC, et al. Daratumumab-Bortezomib-Thalidomide-Dexamethasone for Newly Diagnosed Myeloma: Cassiopeia Minimal Residual Disease Results. *Blood* (2025) 146(6):679‐92. doi: 10.1182/blood.2024027620.

30.	Dimopoulos M, Quach H, Mateos MV, Landgren O, Leleu X, Siegel D, et al. Carfilzomib, Dexamethasone, and Daratumumab Versus Carfilzomib and Dexamethasone for Patients with Relapsed or Refractory Multiple Myeloma (Candor): Results from a Randomised, Multicentre, Open-Label, Phase 3 Study. *Lancet (london, england)* (2020) 396(10245):186‐97. doi: 10.1016/S0140-6736(20)30734-0.

31.	Dimopoulos MA, San-Miguel J, Belch A, White D, Benboubker L, Cook G, et al. Daratumumab Plus Lenalidomide and Dexamethasone Versus Lenalidomide and Dexamethasone in Relapsed or Refractory Multiple Myeloma: Updated Analysis of Pollux. *Haematologica* (2018) 103(12):2088‐96. doi: 10.3324/haematol.2018.194282.

32.	Facon T, Kumar S, Plesner T, Orlowski RZ, Moreau P, Bahlis N, et al. Daratumumab Plus Lenalidomide and Dexamethasone for Untreated Myeloma. *New England journal of medicine* (2019) 380(22):2104‐15. doi: 10.1056/NEJMoa1817249.

33.	Fu W, Bang SM, Huang H, Kim K, Li W, An G, et al. Bortezomib, Melphalan, and Prednisone with or without Daratumumab in Transplant-Ineligible Asian Patients with Newly Diagnosed Multiple Myeloma: The Phase 3 Octans Study. *Clinical lymphoma, myeloma & leukemia* (2023) 23(6):446‐55.e4. doi: 10.1016/j.clml.2023.02.009.

34.	Landgren O, Weisel K, Rosinol L, Touzeau C, Turgut M, Hajek R, et al. Subgroup Analysis Based on Cytogenetic Risk in Patients with Relapsed or Refractory Multiple Myeloma in the Candor Study. *British journal of haematology* (2022) 198(6):988-93. doi: 10.1111/bjh.18233.

35.	Lu J, Fu W, Li W, Hu J, An G, Wang Y, et al. Daratumumab, Bortezomib, and Dexamethasone Versus Bortezomib and Dexamethasone in Chinese Patients with Relapsed or Refractory Multiple Myeloma: Phase 3 Lepus (Mmy3009) Study. *Clinical lymphoma, myeloma & leukemia* (2021) 21(9):e699‐e709. doi: 10.1016/j.clml.2021.04.012.

36.	Mateos MV, Sonneveld P, Hungria V, Nooka AK, Estell JA, Barreto W, et al. Daratumumab, Bortezomib, and Dexamethasone Versus Bortezomib and Dexamethasone in Patients with Previously Treated Multiple Myeloma: Three-Year Follow-up of Castor. *Clinical Lymphoma, Myeloma and Leukemia* (2020) 20(8):509-18. doi: 10.1016/j.clml.2019.09.623.

37.	Moreau P, Hulin C, Perrot A, Arnulf B, Belhadj K, Benboubker L, et al. Maintenance with Daratumumab or Observation Following Treatment with Bortezomib, Thalidomide, and Dexamethasone with or without Daratumumab and Autologous Stem-Cell Transplant in Patients with Newly Diagnosed Multiple Myeloma (Cassiopeia): An Open-Label, Randomised, Phase 3 Trial. *The lancet Oncology* (2021) 22(10):1378‐90. doi: 10.1016/S1470-2045(21)00428-9.

38.	Moreau P, Hulin C, Perrot A, Arnulf B, Belhadj K, Benboubker L, et al. Bortezomib, Thalidomide, and Dexamethasone with or without Daratumumab and Followed by Daratumumab Maintenance or Observation in Transplant-Eligible Newly Diagnosed Multiple Myeloma: Long-Term Follow-up of the Cassiopeia Randomised Controlled Phase 3 Trial. *Lancet oncology* (2024) 25(8):1003‐14. doi: 10.1016/S1470-2045(24)00282-1.

39.	Spencer A, Lentzsch S, Weisel K, Avet-Loiseau H, Mark TM, Spicka I, et al. Daratumumab Plus Bortezomib and Dexamethasone Versus Bortezomib and Dexamethasone in Relapsed or Refractory Multiple Myeloma: Updated Analysis of Castor. *Haematologica* (2018) 103(12):2079‐87. doi: 10.3324/haematol.2018.194118.

40.	Suzuki K, Min CK, Kim K, Lee JJ, Shibayama H, Ko PS, et al. Carfilzomib, Dexamethasone, and Daratumumab in Asian Patients with Relapsed or Refractory Multiple Myeloma: Post Hoc Subgroup Analysis of the Phase 3 Candor Trial. *International journal of hematology* (2021) 114(6):653‐63. doi: 10.1007/s12185-021-03204-9.

41.	Voorhees PM, Sborov DW, Laubach J, Kaufman JL, Reeves B, Rodriguez C, et al. Addition of Daratumumab to Lenalidomide, Bortezomib, and Dexamethasone for Transplantation-Eligible Patients with Newly Diagnosed Multiple Myeloma (Griffin): Final Analysis of an Open-Label, Randomised, Phase 2 Trial. *The Lancet Haematology* (2023) 10(10):e825‐e37. doi: 10.1016/S2352-3026(23)00217-X.

42.	Magrangeas F, Guérin-Charbonnel C, Bessonneau-Gaborit V, Denoulet M, Giordano N, Perrot A, et al. Expression Profile of Cassiopeia Patients Refines Prognostic Value of Mrd Negativity in Multiple Myeloma. *Blood cancer journal* (2026). doi: 10.1038/s41408-026-01550-7.

43.	Suzuki K, Matsumoto M, Takamatsu H, Kosugi H, Kondo T, Fujisaki T, et al. Daratumumab Plus Vrd in Japanese Transplant-Ineligible/Deferred Ndmm Patients: Japanese Subgroup of the Cepheus Trial. *International journal of hematology* (2026) 124(1):104‐13. doi: 10.1007/s12185-026-04185-3.

44.	Zweegman S, Facon T, Hungria V, Bahlis NJ, Venner CP, Braunstein M, et al. Daratumumab in Transplant-Ineligible or -Deferred Newly Diagnosed Multiple Myeloma: Minimal Residual Disease in Cepheus. *Blood advances* (2026). doi: 10.1182/bloodadvances.2026019937.

Supplemental Table 1 The high-risk cytogenetic abnormalities (HRCAs) reported in the included studies

| Study | HRCAs definition | Reported results | | | |
| --- | --- | --- | --- | --- | --- |
|  |  | Revised  HRCAs | One  HRCA | gain/amp  (1q21) | ≥2  HRCAs |
| Bahlis et al.,2025 | del(17p), t(4;14), and/or t(14;16) | No | No | No | No |
| Chari et al.,2024 | del(17p), t(4;14), t(14;16), t(14;20),  and/or gain/amp(1q21) | Yes | Yes | Yes | Yes |
| Dimopoulos et al.,2021 | del(17p), t(4;14), or t(14;16) | No | No | No | No |
| Dimopoulos et al.,2023 | del(17p), t(4;14), or t(14;16) | No | No | No | No |
| Facon et al.,2021 | del(17p), t(4;14), or t(14;16) | No | No | No | No |
| Foster et al.,2025 | del(17p), t(4;14), t(14;16), t(14;20),  and/or gain/amp(1q21) | Yes | Yes | Yes | Yes |
| Fu et al.,2023 | del(17p), t(4;14), or t(14;16) | No | No | No | No |
| Fu et al.,2025 | del(17p), t(4;14), t(14;16), t(14;20),  and/or gain/amp(1q21) | Yes | Yes | Yes | Yes |
| Kaufman et al.,2020 | del(17p), t(4;14), or t(14;16) | No | No | No | No |
| Mateos et al.,2018 | del(17p), t(4;14), or t(14;16) | No | No | No | No |
| Mateos et al.,2025 | del(17p), t(4;14), or t(14;16) | No | No | No | No |
| Mollee et al.,2024 | del(17p), t(4;14), or t(14;16) | No | No | No | No |
| Moreau et al.,2019 | del(17p) or t(4;14) | No | No | No | No |
| Moreau et al.,2025 | del(17p), t(4;14), t(14;16), t(14;20),  and/or gain/amp(1q21) | Yes | Yes | Yes | Yes |
| Sonneveld et al.,2023 | del(17p), t(4;14), or t(14;16) | No | No | No | No |
| Sonneveld et al.,2024 | del(17p), t(4;14), or t(14;16) | No | No | No | No |
| Usmani et al.,2022 | del(17p), t(4;14), or t(14;16) | No | No | No | No |
| Usmani et al.,2023 | del(17p), t(4;14), or t(14;16) | No | No | No | No |
| Usmani et al.,2025 | del(17p), t(4;14), or t(14;16) | No | No | No | No |
| Weisel et al.,2020 | del(17p), t(4;14), or t(14;16) | No | No | No | No |

Supplemental Figure 1: Risk of bias evaluation of the included randomized controlled trials.

Supplemental Figure 2: Sensitivity analysis of the effect of daratumumab on progression-free survival.

Supplemental Figure 3: Subgroup analysis according to daratumumab administration methods (Progression-free survival).

Supplemental Figure 4: Subgroup analysis according to follow-up duration (Progression-free survival).

Supplemental Figure 5: Sensitivity analysis of the effect of daratumumab on patients with revised cytogenetic risk (Progression-free survival).

Supplemental Figure 6: Sensitivity analysis of the effect of daratumumab on overall survival.

Supplemental Figure 7: Subgroup analysis according to multiple myeloma subtypes (Overall survival).

Supplemental Figure 8: The funnel plots of the effect of daratumumab on progression-free survival.

Supplemental Figure 9: The Egger’s test of the effect of daratumumab on progression-free survival.

Supplemental Figure 10: The Bgger’s test of the effect of daratumumab on progression-free survival.
